# Systemic sclerosis with morphea-like plaques histopathologically mimicking cutaneous B-cell lymphoma

**DOI:** 10.1016/j.jdcr.2024.02.028

**Published:** 2024-03-08

**Authors:** Ana M. Aragon Sierra, Angelina S. Hwang, Jacob Kechter, Aaron R. Mangold, Vivek Nagaraja, David J. DiCaudo

**Affiliations:** a Mayo Clinic Alix School of Medicine, Mayo Clinic, Scottsdale, Arizona; b Department of Dermatology, Mayo Clinic, Scottsdale, Arizona; c Division of Rheumatology, Department of Internal Medicine, Mayo Clinic, Scottsdale, Arizona; d Department of Laboratory Medicine and Pathology, Mayo Clinic, Scottsdale, Arizona

**Keywords:** autoimmune disease, B-cell lymphoma, morphea, pseudolymphoma, scleroderma, systemic sclerosis

## Introduction

Systemic sclerosis and morphea share a similar histopathologic pattern on skin biopsy, but the 2 entities differ in their clinical manifestations. Common cutaneous findings in systemic sclerosis include sclerodactyly, Raynaud phenomenon, fingertip lesions, and telangiectasias. By contrast, morphea presents with localized or generalized indurated cutaneous plaques. In rare cases, features of morphea and systemic sclerosis may coexist in the same patient.^1^^,^^2^

The characteristic histopathologic feature of both systemic sclerosis and morphea is dermal sclerosis. Lymphoid follicles are not a typical feature but have been rarely reported in association with dermal sclerosis in skin biopsies of morphea.^3^^,^^4^ We report a case of systemic sclerosis presenting with morphea-like plaques and prominent lymphoid follicles on skin biopsy. These findings were misdiagnosed initially as cutaneous B-cell lymphoma. This case highlights both an unusual clinical presentation of systemic sclerosis and a potential histopathologic pitfall of lymphoid follicles in biopsies of sclerosing disorders.

## Case report

A 62-year-old female presented to a local dermatology practice for a 3-month history of indurated plaques on the upper chest with progressive skin erythema and hardening. She also experienced decreased range of motion in the shoulders secondary to skin tightness. The patient was treated with hydroxychloroquine and topical clobetasol without benefit for a suspected diagnosis of morphea profunda. Skin biopsy was interpreted as an atypical lymphoid infiltrate, and a diagnosis of B-cell lymphoma was favored by the outside pathologist. Subsequent evaluation by a local physician included a negative computed tomography scan of the chest, abdomen, and pelvis and negative peripheral blood flow cytometry. The patient was then referred to our institution for consultation.

**Fig 1:**
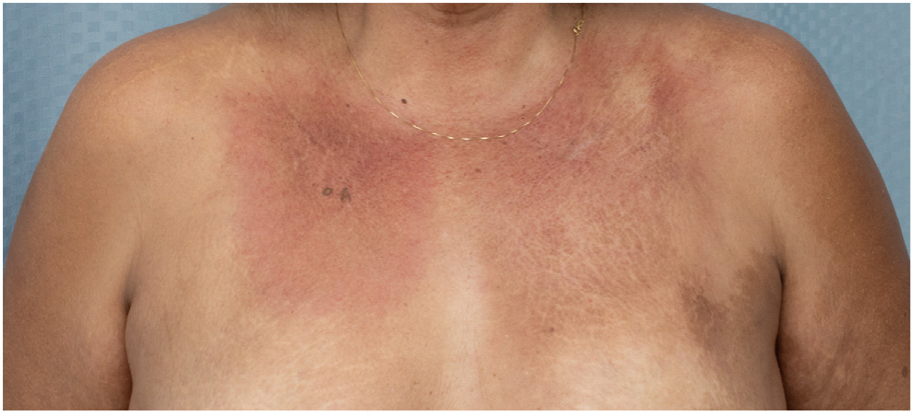
Indurated violaceous plaques on the upper chest.

**Fig 2:**
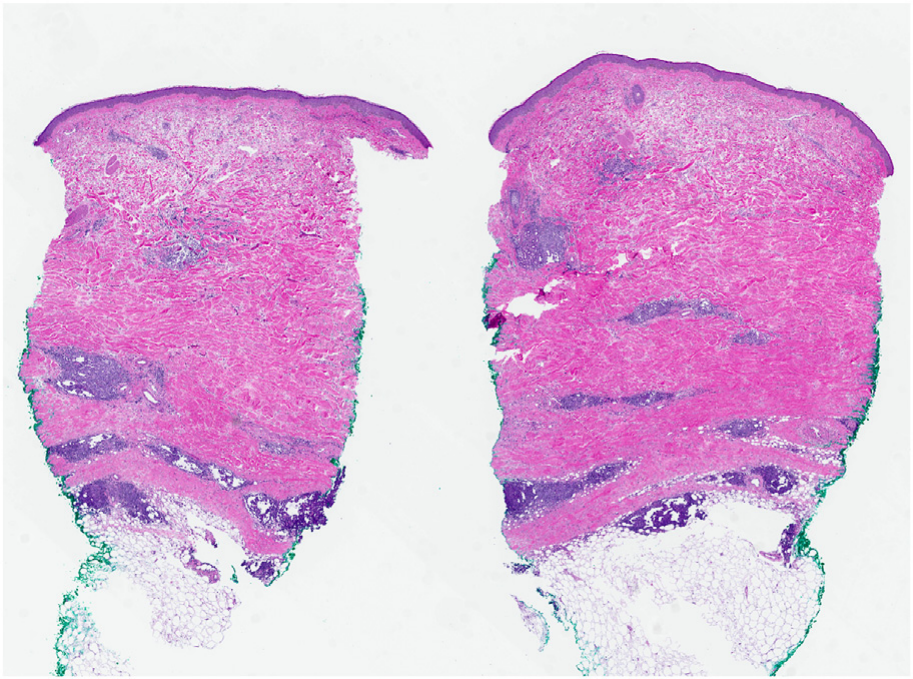
Rectangular biopsy specimen with compact brightly eosinophilic dermal collagen and scattered lymphoid nodules. Hematoxylin-eosin, magnification × 1 from digital scan.

**Fig 3:**
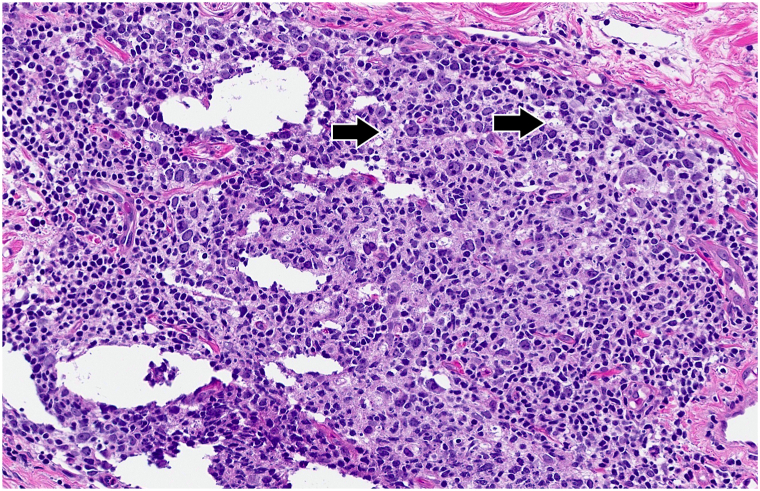
Reactive dermal lymphoid follicle with many tingible body macrophages (*arrows*). Hematoxylin-eosin, magnification × 16 from digital scan.

**Fig 4:**
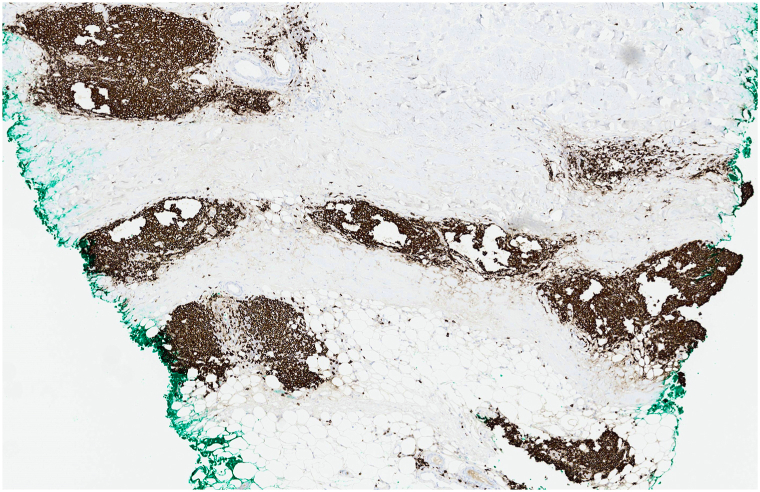
Diffuse expression of the B-cell marker CD20 throughout the infiltrate (×3 from digital scan).

On physical examination, the patient had indurated violaceous plaques on the upper chest involving 6% body surface area (Fig 1). A repeat punch biopsy from the chest revealed a rectangular-shaped specimen with diffuse sclerosis of the dermal collagen (Fig 2). Among the sclerotic collagen bundles, there were many scattered lymphoid follicles with tingible body macrophages (Fig 3). The CD34 immunostain revealed markedly diminished dendrocytes in the reticular dermis. CD20 was positive in the lymphoid follicles (Fig 4), and CD21 highlighted intact follicular dendritic cell networks. BCL6-positive follicle center cells were confined to the lymphoid follicles. Ki67 was expressed by 75% of cells within the follicle centers. Plasma cells at the periphery of the follicles had polytypic light chain expression. The overall findings supported the diagnosis of morphea with reactive lymphoid follicles. The original skin biopsy specimen, which initially had been suspected to show B-cell lymphoma, was reviewed by another local dermatopathology consultant, who similarly favored a diagnosis of morphea for the original skin biopsy specimen. Serological evaluation was notable for an elevated anti-nuclear antibody of 6.6 units (normal <1.0 units) and positive anticentromere antibodies (>8.0; normal <1.0 units). Antidouble-stranded DNA, anti-Ro (SS-A), anti-La (SS-B), anti-Smith, anti-ribonucleoprotein, anti-Scl 70, anti-Jo 1, and antiribosomal P antibodies were negative. Laboratory results for renal and liver function were unremarkable.

On evaluation by the rheumatologist, the patient had dilated nailfold capillaries with occasional microhemorrhages and telangiectasias on the face and the oral mucosa. She reported dysphagia, acid-reflux symptoms, and polyarthralgia involving the hips, low back, neck, and shoulders. Interestingly, she did not have sclerodactyly and denied any Raynaud symptoms. A diagnosis of systemic sclerosis was made based on the overall findings. The patient was initiated on methotrexate 15 mg by mouth weekly for immunosuppressive therapy. She experienced decreased joint symptoms and remarkable improvement of sclerodermatous changes on the chest within 1 month.

## Discussion

This case of systemic sclerosis is remarkable both for the morphea-like plaques noted clinically and the prominent B-cell follicles seen histopathologically. Morphea-like lesions have rarely been reported in patients with systemic sclerosis. Soma et al reported that 6.7% of their patients with systemic sclerosis also had morphea.^5^ Chen et al found that 3.2% of their patients had coexisting systemic sclerosis and morphea.^2^ In our patient, the combined findings of dilated nailfold capillaries, telangiectasias, dysphagia, and anticentromere antibodies supported the diagnosis of systemic sclerosis despite the absence of sclerodactyly and the absence of Raynaud phenomenon.

An additional remarkable feature of this case is the histopathologic finding of prominent lymphoid follicles within the sclerotic dermis. This is an especially unusual finding in systemic sclerosis, which typically has only minimal inflammation. Perivascular infiltrates of lymphocytes and plasma cells are common in morphea and may be prominent. Yet, even in inflammatory morphea, lymphoid follicles have only rarely been reported.^3^ In a recent review of 137 cases of morphea, the lymphocytic infiltrates were variably distributed in patterns that were perivascular or periadnexal or at the dermal-subcutaneous interface. Lymphoid follicles are not specifically described in this large case series.^6^ The prominent lymphoid follicles in this case initially led to consideration of cutaneous B-cell lymphoma. However, in our own biopsy specimen, the lymphoid follicles had reactive histopathologic features including the presence of tingible body macrophages and a high proliferative index with Ki67, as typically seen in reactive follicular hyperplasia. In addition, the BCL6-positive follicle center cells were confined to the lymphoid follicles, as outlined by the intact CD21-positive follicular dendritic cell networks. These features are typical of benign reactive follicular lymphoid hyperplasia and contrast with the expected findings in neoplastic lymphoid follicles.^7^

Although inflammatory morphea is recognized as potential mimicker of cutaneous T-cell lymphoma, the literature does not emphasize the potential for misdiagnosis of cutaneous B-cell lymphoma in patients with morphea or systemic sclerosis.^8^, ^9^, ^10^ Recognition of the rare finding may assist in avoiding a diagnostic pitfall.

## Conflicts of interest

Dr Mangold has no conflicts of interest directly related to this report. Dr. Mangold has received grants/research fundings from Kyowa, Miragen, Regeneron, Corbus, Sun Pharma, Incyte, Pfizer Inc, Merck & Co, Priovant, Eli Lilly, Elorac, Novartis, Janssen, Soligenix, Argenix, Palvella, and Abbvie; and has served as a consultant for Kyowa, Eli Lilly, Momenta, UCB, Regeneron, Incyte, PHELEC, Soligenix, Clarivate, Argenyx, Janssen, Bristol-Myers Squibb, Boehringer Ingelheim, and Pfizer. He has 2 provisional IP/patents and 1 filed IP/patent, that is, Methods and Materials for Assessing and Treating Cutaneous Squamous Cell Carcinoma-provisional 63-423254; Use of Oral Jaki in Lichen Planus-provisional 63/453,065; and Topical Ruxolitinib in Lichen Planus-wo2022072814a1, respectively. Author Aragon Sierra, Author Hwang, Author Kechter, Dr Nagaraja, and Dr DiCaudo have no conflicts of interest to declare.
